# Obstacle Detection Method Based on RSU and Vehicle Camera Fusion

**DOI:** 10.3390/s23104920

**Published:** 2023-05-19

**Authors:** Shaohong Ding, Yi Xu, Qian Zhang, Jinxin Yu, Teng Sun, Juan Ni, Shuyue Shi, Xiangcun Kong, Ruoyu Zhu, Liming Wang, Pengwei Wang

**Affiliations:** 1 School of Transportation and Vehicle Engineering, Shandong University of Technology, Zibo 255000, China; 21402030147@stumail.sdut.edu.cn (S.D.); 21502050244@stumail.sdut.edu.cn (J.Y.); 21502050238@stumail.sdut.edu.cn (T.S.); 22502060003@stumail.sdut.edu.cn (J.N.); 22502060007@stumail.sdut.edu.cn (S.S.); 22502060006@stumail.sdut.edu.cn (X.K.); 20502040227@stumail.sdut.edu.cn (R.Z.); 20402010134@stumail.sdut.edu.cn (L.W.); wangpw@sdut.edu.cn (P.W.); 2 Collaborative Innovation Center of New Energy Automotive, Shandong University of Technology, Zibo 255000, China; 3 Jinan Engineering Polytechnic, Jinan 250200, China; jngczyjsxyjwc@jn.shandong.cn

**Keywords:** obstacle detection, road-side unit, inertial measuring unit, maximally stable extremal regions, User Data Protocol

## Abstract

Road obstacle detection is an important component of intelligent assisted driving technology. Existing obstacle detection methods ignore the important direction of generalized obstacle detection. This paper proposes an obstacle detection method based on the fusion of roadside units and vehicle mounted cameras and illustrates the feasibility of a combined monocular camera inertial measurement unit (IMU) and roadside unit (RSU) detection method. A generalized obstacle detection method based on vision IMU is combined with a roadside unit obstacle detection method based on a background difference method to achieve generalized obstacle classification while reducing the spatial complexity of the detection area. In the generalized obstacle recognition stage, a VIDAR (Vision-IMU based identification and ranging) -based generalized obstacle recognition method is proposed. The problem of the low accuracy of obstacle information acquisition in the driving environment where generalized obstacles exist is solved. For generalized obstacles that cannot be detected by the roadside unit, VIDAR obstacle detection is performed on the target generalized obstacles through the vehicle terminal camera, and the detection result information is transmitted to the roadside device terminal through the UDP (User Data Protocol) protocol to achieve obstacle recognition and pseudo-obstacle removal, thereby reducing the error recognition rate of generalized obstacles. In this paper, pseudo-obstacles, obstacles with a certain height less than the maximum passing height of the vehicle, and obstacles with a height greater than the maximum passing height of the vehicle are defined as generalized obstacles. Pseudo-obstacles refer to non-height objects that appear to be “patches” on the imaging interface obtained by visual sensors and obstacles with a height less than the maximum passing height of the vehicle. VIDAR is a vision-IMU-based detection and ranging method. IMU is used to obtain the distance and pose of the camera movement, and through the inverse perspective transformation, it can calculate the height of the object in the image. The VIDAR-based obstacle detection method, the roadside unit-based obstacle detection method, YOLOv5 (You Only Look Once version 5), and the method proposed in this paper were applied to outdoor comparison experiments. The results show that the accuracy of the method is improved by 2.3%, 17.4%, and 1.8%, respectively, compared with the other four methods. Compared with the roadside unit obstacle detection method, the speed of obstacle detection is improved by 1.1%. The experimental results show that the method can expand the detection range of road vehicles based on the vehicle obstacle detection method and can quickly and effectively eliminate false obstacle information on the road.

## 1. Introduction

With the development of image technology and the improvement of information acquisition methods, vision-based obstacle recognition methods are gradually becoming the main trend in obstacle recognition. Many researchers and companies have developed obstacle recognition methods using cameras. In recent years, intelligent network technology has developed rapidly, and a large number of roadside unit devices are used in important places, such as highways as well as urban roads. In practical applications, roadside units are used as traffic event recording devices to assist manual monitoring as well as to provide over-the-horizon road information to vehicles. When distinguishing obstacle categories, they cannot identify some broad obstacles, such as wooden boards, plastic bags, etc. Extracting broad obstacle information from surveillance video has become an important problem to be solved in the current traffic field.

Driving environment detection is a key technology to achieve vehicle obstacle avoidance. The purpose of traveling environment detection is to provide data support for the subsequent vehicle driving strategy by obtaining information about the surrounding environment. Roadside units occupy an important position in the intelligent transportation system, and the research on obstacle detection of roadside units is also a popular research topic today. In this paper, based on the generalized obstacle detection method of VIDAR [1], a generalized obstacle identification method applicable to roadside units is proposed. Based on this, an effective communication protocol is constructed by using communication technology to combine machine vision-based generalized obstacle detection with roadside units to achieve the accurate representation of road obstacle information. The method can detect a generalized obstacle that cannot be discerned by a roadside unit using a vehicle-mounted camera. The obstacle is classified by the height of the generalized obstacle, and its height information is transmitted to the roadside unit end, thus providing the vehicle with overview distance. This method makes up for the shortcoming of the roadside unit not being able to recognize generalized obstacles and also solves the problem of the sight distance defect of the vehicle-mounted camera.

The rest of this article is organized as follows. In Section 2, we describe the related research on obstacle detection and communication between roadside equipment and vehicle equipment. Section 3 introduces the obstacle extraction method of the RSU (Section 3.1), focusing on the vehicle-side obstacle detection method based on VIDAR (Section 3.2) and the design of the data interaction method at both ends (Section 3.3). Section 4 gives the experimental results of obstacle detection and the feasibility analysis of data interaction. In Section 5, we analyze the effect of the generalized obstacle detection method based on the fusion of RSU and the vehicle camera. By comparing the obstacle recognition method based on VIDAR and the obstacle detection method based on RSU, the superiority of the method is obtained. In Section 6, the overall overview and shortcomings of this paper are summarized, which provides direction for subsequent research.

## 2. Related Works

Obstacle detection, as a core aspect of advanced assisted driving for vehicles and other fields, is a popular research problem today. Additionally, in obstacle detection, monocular vision has become one of the main research directions for obstacle detection today due to its low cost and relatively simple measurement principle. In order to improve the accuracy of obstacle detection, a large number of scholars and manufacturers at home and abroad have carried out a significant amount of relevant research. Sabir Z. et al. [2] This paper proposes a communication strategy that incorporates a vehicle factor. This approach provides the driver with risk information about the environment at the appropriate time, and the vehicle owner can choose a navigation option based on the warning information. Finally, the communication function between the roadside unit and the vehicle is implemented through a Wi-Fi based application. However, this paper only tells the process of roadside units to identify obstacles at the broad level and does not consider the situation when the road appears as a broad obstacle. Rateke et al. [3] used convolutional neural networks for obstacle detection. The CNN (Convolutional Neural Networks)-based detection and target recognition results were combined with the depth pattern of the parallax map and the motion pattern of the optical flow in the validation phase. Song et al. [4] proposed a refinement module based on a stacked hourglass network with improved predicted semantic segmentation and parallax mapping. The method outperforms existing semantic segmentation networks in road obstacle detection and can be used for unlabeled obstacles. Gholami et al. [5] used a real-time obstacle detection method based on stereo vision and ultrasonic data fusion to detect stereo obstacle information. Weon et al. [6] used a 3D random sample consensus algorithm to extract ground data perpendicular to the reference estimated 3D plane and data at both ends by ground estimation by matching the marker information of the defined objects with the classified object cloud data obtained using 3D LiDAR (Light detection and ranging). The method efficiently processes the collected object data while maintaining a high detection rate. Bansal et al. [7] acquired the height, width, and slope of adjacent vertices in the image by CCD (Charge Coupled Device) camera and low cost sensors for target detection in 3D space. Xu et al. [8] installed a four-way fisheye camera in a car and used the acquired environmental images as input. The YOLOv3 network structure was used to detect parking spaces in the images. This algorithm can also be applied to obstacle detection with a high check-all rate and detection accuracy for real-time detection. However, these studies do not take into account the presence of generalized obstacles in the driving environment. Huu et al. [9] proposed a machine-vision-based two-lane detection algorithm that uses a pre-trained YOLO dataset to identify obstacle information in images detected by the vehicle’s front camera. Each target bounding box of the network is represented by a vector. By setting the box probability threshold, the boxes with a lower probability are removed, thus achieving the screening and rejection of obstacle information. Dhouioui et al. [10] proposed an embedded system based on two types of data, i.e., radar signals and camera images, to identify and classify obstacles on the road. The method extracts features from the road information data collected in the sensors to obtain the feature vectors of the obstacles. A training machine learning model was used as the model in the image processing stage. The overall computational performance and efficiency of obstacle recognition was optimized by machine learning methods and signal processing techniques. Xue et al. [11] proposed an improved YOLOv5s algorithm based on k-means clustering and the CIoU (Complete Intersection over Union) loss function. The k-means clustering algorithm was used to generate the initial marker frame size of the target obstacle to accelerate the convergence speed of model training. Combining three geometric measures of the obstacle marker frame overlap area, center distance, and aspect ratio reduced the obstacle miss and false detection rate and improved the detection accuracy. Sengar et al. [12] proposed a method to detect moving objects using frame differencing. In order to meet the requirement of obstacle detection in a driving environment where generalized obstacles exist, Jiang et al. [13,14] proposed a VIDAR-based generalized obstacle detection method. The case of daytime obstacle detection using VIDAR and a machine learning algorithm jointly detected the reflection of unknown obstacles at nighttime, using the improved VIDAR for obstacle detection. The method has been improved in terms of the accuracy of obstacle detection. The current obstacle detection method has a high detection speed and accuracy for common general obstacles such as vehicles, but the false detection rate is high for the driving environment with unknown obstacles, especially in the driving environment where obstacles such as paper, plastic bags, and wooden boards exist.

For obstacle detection at the side unit end, Steinbaeck et al. [15] built an electric unmanned vehicle consisting of an ROS (Robot Operating System) architecture and proposed a V2X (vehicle to everything)-based vehicle localization method. A sensor fusion method for obstacle detection by radar and optical range information, the proposed method can improve the localization accuracy by more than 60% compared to using GPS (Global Positioning System) only. However, the error between the detected obstacle position result and its real position is large, which cannot be applied to some scenarios with high accuracy. Additionally, the vehicle positioning deviation will occur in an environment with a poor GPS signal. Mouawad et al. [16] improved the safety of C-ITS (Cooperative Intelligent Transportation System) vulnerable road users by considering a cooperative collision avoidance system. In this system, the RSU placed in the center of the intersection can fuse continuously receiving maps, forming a global occupied map. Vehicles rely on onboard sensors to generate local occupied maps. This is transmitted to a fusion center via an LTE-V2X connection, thus enabling real-time access to environmental information. However, the method does not consider the presence of broad obstacle situations on the road and lacks real experimental data support.

**Figure 1:**
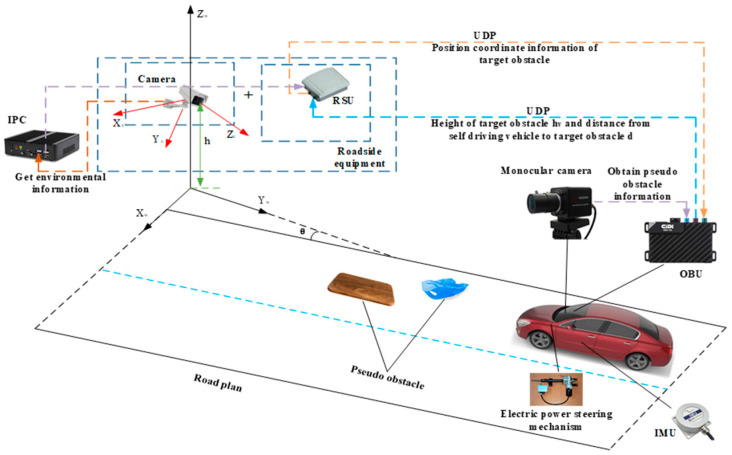
Schematic diagram of obstacle detection model based on RSU and vehicle camera fusion.

For the communication between roadside units and vehicles, Ghovanlooy Ghajar et al. [17] proposed a blockchain-based scalable trust management system for VANET (Vehicular Ad-hoc NETwork). This system provides the vehicle with its surrounding driving environment and evaluates the reliability of the information about the surrounding environment acquired by the vehicle through its own sensors. AĞGÜN et al. [18] proposed a new reservation-based multichannel hybrid MAC protocol, ReMAC. This protocol provides an efficient channel allocation mechanism and reduces the time of network access delay. In this paper, due to the small amount of data to be transmitted, the UDP protocol is used to implement the environmental information transmission between the OBU (On board Unit) and RSU. Figure 1 shows the obstacle detection model with the fusion of the roadside unit and on-board camera.

Combined with the above literature, this paper proposes a generalized obstacle detection method based on the fusion of the roadside unit and vehicle-mounted camera. The obstacle detection method of visual IMU is applied in the extracted background area to detect the unknown type of obstacles. The generalized obstacles are detected and rejected according to the VIDAR generalized obstacle detection principle, and the identified pseudo-obstacles and the generalized obstacles with low height are removed by measuring the distance and speed of the non-generalized obstacles, thus providing a good data base for the control strategy. In this paper, a camera is added at the roadside unit end, the broad field of view of the roadside unit is used to identify the generalized obstacles in the environment by the background difference method, and the location of the generalized obstacles that cannot be identified is marked. The joint VIDAR-based vehicle-side obstacle detection method is used when pseudo-obstacles enter the vehicle-side machine vision detection range. The height of the generalized obstacle is used to judge the authenticity of the obstacle, and the 3D information of the obstacle is marked in the processing interface. Finally, the information is stored in the roadside device for the required vehicle. This method, on the one hand, solves the problem of vehicle positioning deviation due to poor signal when using GPS and, on the other hand, solves the problem of the difficult detection of generalized obstacles in the road.

## 3. Method

**Figure 2:**
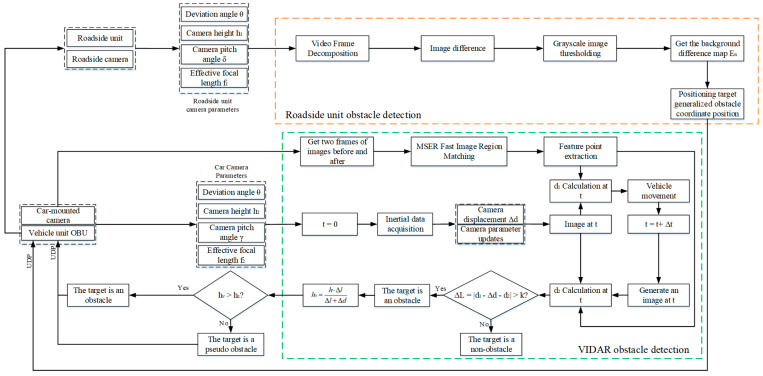
Obstacle detection based on fusion of RSU and vehicle camera.

For the roadside unit detection environment view that cannot detect generalized obstacles which are near two-dimensional objects, this paper proposes an obstacle detection method based on the fusion of the roadside unit and vehicle-mounted camera. The roadside unit camera is used to obtain the road environment information (including the localization of generalized obstacles, the relative position relationship between obstacles, etc.). For the generalized obstacles that cannot be recognized by the roadside unit side, the obstacle detection method based on VIDAR can be used by the vehicle-side camera to determine the authenticity of the obstacles based on the height of the generalized obstacles. The vehicle-end camera can also detect the distance information before the self-vehicle and the target obstacle. Through the UDP protocol, the generalized obstacle authenticity and distance information are transmitted to the roadside unit end to improve the road environment information at the roadside unit end and provide the overview distance for subsequent vehicles. At present, the commonly used obstacle detection method is to obtain the camera motion parameters by using the sensor or motion estimation method and achieve obstacle detection by compensating for the camera motion [19,20,21]. However, this method is less efficient and has certain errors in practical operation. In this paper, the generalized obstacle information acquired by machine vision is transmitted to RSU, and the data processing operation is completed in the computational unit of RSU. This method greatly improves the data processing efficiency and reserves time redundancy for subsequent operations, such as vehicle path planning and obstacle avoidance. The specific process of obstacle detection by roadside unit and vehicle camera fusion is shown in Figure 2.

### 3.1. RSU Obstacle Extraction

**Figure 3:**
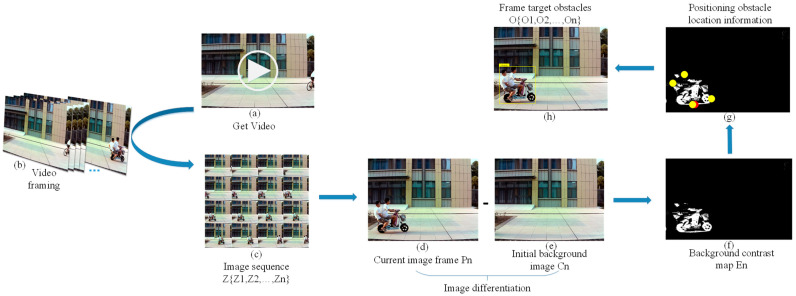
Obstacle extraction based on background subtraction method, where (**a**) is the acquired video; (**b**) is the video framing result map; (**c**) is the image sequence map after video framing processing; (**d**) is the current frame image; (**e**) is the initial background map; (**f**) is the obstacle region acquired by background subtraction method processing of the comparison image; (**g**) is the coordinate position acquisition of this obstacle, where ● is the coordinate point corresponding to the minimum horizontal coordinate, maximum horizontal coordinate, minimum vertical coordinate, and maximum vertical coordinate of the obstacle, respectively; ● is the position coordinate of the obstacle; and (**h**) is the position region acquisition of the obstacle.

**Figure 4:**
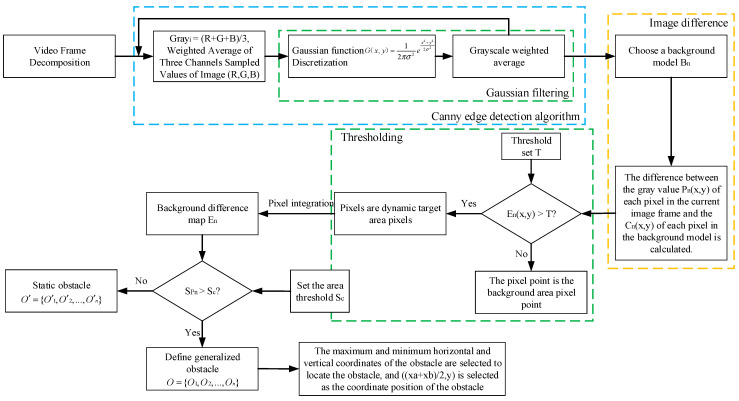
Flowchart of RSU obstacle extraction method based on background subtraction method.

The background difference method is widely used in dynamic obstacle extraction [22,23,24,25,26,27,28,29]. In this paper, a roadside unit obstacle extraction method based on the background difference method is used. Firstly, two frames detected by the roadside unit camera are selected (one frame is the initial background image with no broad obstacle set for the road, and the other frame is the current frame image with the broad obstacle present), and the pixel threshold is preset. The current frame image and the initial background image are differenced to exceed the pixel threshold to define the motion region, which is the generalized obstacle $O=\{O_{1},O_{2},\ldots ,O_{n}\}$. The general detection steps are shown in Figure 3:

Since the detection object of this method is the global region of the image, the detected dynamic target result is more reliable, and the generalized obstacles that do not appear in the initial background image can be detected and eliminated. The coordinates of the obstacle in the scene $\left(x_{i},y_{j}\right)$ are obtained by converting the image coordinates fused by the roadside unit and the on-board camera to the world coordinate system. The specific process is as follows:

1. Video framing. In order to select the test scene suitable for the establishment of this paper, the video frame should be decomposed, and the video image should be decomposed into an image sequence $Z=\{Z_{1},Z_{2},\ldots ,Z_{n}\}$ according to a certain time or period.
2. Image preprocessing. All channel sampling values of each image in image sequence $Z=\{Z_{1},Z_{2},\ldots ,Z_{n}\}$ are weighted average, that is, the image is grayed. Then, the Gaussian function $G(x,y)=\frac{1}{2\pi \sigma^{2}}e^{\frac{x^{2}+y^{2}}{2\sigma^{2}}}$ is discretized. The Gaussian function value G(x,y) is used as the weight, and the pixels in the grayscale image are weighted and averaged to suppress the noise in the image. In order to make the edge contour in the image smoother, the pre-processed image is closed to solve the adaptive repair of contour fracture in the gray image.
3. Build background model $C_{n}$. According to the image sequence $Z=\{Z_{1},Z_{2},\ldots ,Z_{n}\}$ decomposed by step (1), the scene image suitable for the establishment of this paper is selected as the initial background image, that is, the background model $C_{n}$.
4. Calculate image pixel difference value $E_{n}(x,y)$ and image thresholding. The difference between the gray value $P_{n}(x,y)$ of each pixel in the current image frame and the image $C_{n}(x,y)$ of each pixel in the background model is calculated, where $E_{n}(x,y)=P_{n}(x,y)-C_{n}(x,y)$. According to Equation (1), the dynamic pixels in the region are judged by comparing the difference value $E_{n}(x,y)$ of each pixel with the preset threshold T. If the difference value $E_{n}(x,y)$ of the pixel is greater than the threshold T, the pixel is the dynamic target area pixel. If the difference value $E_{n}(x,y)$ of the pixel is less than the threshold T, the pixel is the background area pixel. Finally, the dynamic target area pixels are integrated to obtain the background difference image $E_{n}$:
  (1) $$FZ(x,y)=\left\{\begin{array}{c} 1,E_{n}(x,y)\geq T\\ 0,E_{n}(x,y)<T \end{array}\right.$$
5. Calculate the obstacle position coordinate $\left(x_{i},y_{j}\right)$ and locate the obstacle position. False recognition of dynamic pixels is caused by the influence of external environment (light changes, wind speed, etc.). In this paper, we set the object change area threshold $S_{c}(Z-Z_{i})$ in two frames, where $\left(Z-Z_{i}\right)$ is the period step set by the image sequence. The generalized obstacle $O=\{O_{1},O_{2},\ldots ,O_{n}\}$ is defined by comparing the change area, where this obstacle vertical coordinate $y_{j}$ is the value of the lowest point vertical coordinate of the target obstacle. The obstacle horizontal coordinate $x_{i}$ is the arithmetic mean of the maximum horizontal coordinate value $x_{b}$ and the minimum horizontal coordinate value $x_{a}$ corresponding to the longitudinal coordinate of the lowest point of the target obstacle. Finally, the coordinate points corresponding to the minimum abscissa, the maximum abscissa, the minimum ordinate, and the maximum ordinate of the obstacle in the background difference image $E_{n}$ are extracted. The above four points are used as frames, and the frame area is the obstacle position area. Figure 4 is the overall flow chart of the RSU generalized obstacle extraction.

### 3.2. Vehicle-Side Obstacle Detection Method Based on VIDAR

#### 3.2.1. An Obstacle Region Extraction Method Based on MSER Fast Image Matching

**Figure 5:**
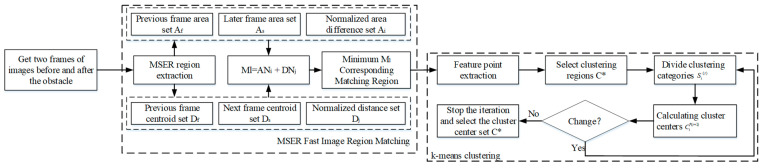
Flowchart of obstacle extraction method based on MSER.

Since the detection object of this method is the regional features of the image rather than the local features, the detected feature points are more stable and can quickly detect the obstacles in the image. In order to determine the location of obstacles in the image, the k-means mean clustering algorithm is used to cluster and analyze the obstacle feature point dataset $C=\{C_{1},C_{2},\ldots ,C_{n}\}$. The specific methodological procedure is as follows [30,31]:

1. MSER algorithm is used to extract the maximum stable extreme value region $Q\_\{i^{*}\}$.
2. Regional range difference $AN_{i}$ is calculated for the two frames of images collected in the experiment. It is assumed that the MSER region sets of the two frames are $Af=\left\{Af_{1},Af_{2},\ldots ,Af_{n}\right\}$ and $As=\left\{As_{1},As_{2},\ldots ,As_{n}\right\}$, respectively.$A_{i}$ is the set of differences between the ith MSER region range in the previous frame and the unmatched region in the next frame. The $A_{i}$ set is normalized, and the effect of normalization is represented by $AN_{i}$. The calculation equation for $AN_{i}$ is
  (2) $$AN_{i}=\frac{Ai-\min(Ai)}{\max(Ai)-\min(Ai)}$$
3. Area set spacing $DN_{j}$ calculation. It is assumed that the centroid sets of MSER regions in the two frames are $Df=\left\{Df_{1},Df_{2},\ldots ,Df_{m}\right\}$ and $Ds=\left\{Ds_{1},Ds_{2},\ldots ,Ds_{m}\right\}$, respectively. $D_{j}$ is the set of distances between the range of the jth MSER region in the previous image and the unmatched region in the subsequent image. The $D_{j}$ set is normalized, and the processing result is represented by $DN_{j}$. The calculation equation for $DN_{j}$ is
  (3) $$DN_{j}=\frac{D_{j}-\min(D_{j})}{\max(D_{j})-\min(D_{j})}$$
4. Extract the matching region $M_{l}$. Let $M_{l}$ be the matching value set of the lth MSER, and extract the MSER corresponding to the minimum $M_{l}$ as the matching region.
5. Object $C^{*}=\{C_{1}^{*},C_{2}^{*},\ldots ,C_{k}^{*}\}$ is selected as the clustering center [32]. Calculate the Euclidean distance between the obstacle feature point and the cluster center according to Equation (4), where $C_{p}$ is the center of clustering at a point, and the region to which $C_{p}$ belongs is divided by calculating the distance between the center point of clustering $C_{p}$ and regions $C_{i}^{*}$ and $C_{l}^{*}$. By setting the distance threshold U, the data whose distance from the clustering center is less than the threshold U are classified into one class:
  (4) $$S_{i}^{\left(t\right)}=\{C_{p}:{\left\|C_{p}-C_{i}^{*(t)}\right\|}^{2}\leq {\left\|C_{p}-C_{l}^{*(t)}\right\|}^{2}\forall l,1\leq l\leq k\}$$
6. According to Equation (5), the cluster center of class i is recalculated:
  (5) $$C_{i}^{*(t+1)}=\frac{1}{{s_{i}}^{\left(t\right)}}\sum_{Cl\in s_{i}^{\left(t\right)}}C_{l}$$
7. The obstacle feature points are iterated according to the repeated steps 5 and 6 until the cluster centroid set $C^{*}=\{C_{1}^{*},C_{2}^{*},\ldots ,C_{k}^{*}\}$ does not change. Figure 5 is the overall flowchart of obstacle extraction.

#### 3.2.2. Static Obstacle Detection

**Figure 6:**
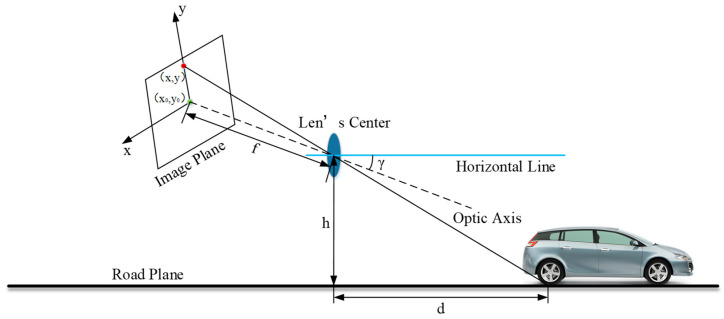
Schematic diagram of pinhole camera mode, where γ is the camera pitch angle, f is the effective focal length of the camera, h is the installation height of the camera, d is the horizontal distance from the camera to the target obstacle.

Obstacle image information acquisition is the process of mapping a target obstacle in a three-dimensional space to a two-dimensional imaging plane, which simplifies the vision sensor that detects generalized obstacle information to a pinhole camera model (see Figure 6). The effective focal length of the camera is f, the installation height of the camera is h, and the pitch angle of the camera is γ. The origin of the two-dimensional imaging plane coordinate system is $\left(x_{0},y_{0}\right)$, and the intersection of the image plane and the central axis of the camera is usually set to $\left(0,0\right)$. The intersection point between the obstacle in front and the road plane is $\left(x,y\right)$, and the coordinate of P point in the image plane coordinate system is $\left(x,y\right)$. The horizontal distance d between the point and the camera is

(6) $$d=\frac{h}{\tan\left(\gamma +\arctan\left[\left(y-y_{0}\right)/f\right]\right)}$$

**Figure 7:**
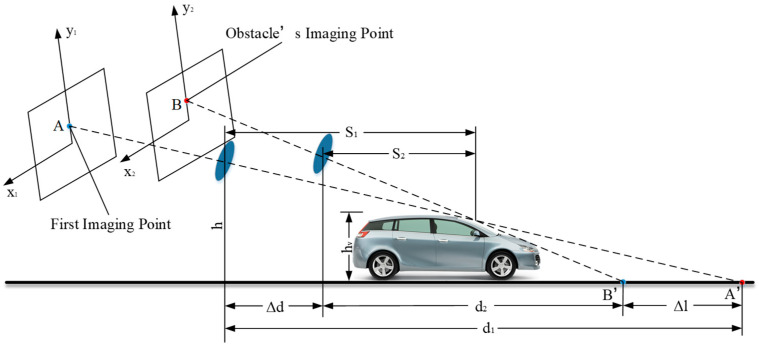
Schematic diagram of static obstacle imaging, where $S_{1}$ is the horizontal distance from the camera to the vertex of the obstacle at the previous moment, $S_{2}$ is the horizontal distance from the camera to the same vertex of the obstacle in the following moment, h is the installation height of the camera, $h_{v}$ is the height of the obstacle, Δd is the distance that the camera moves at two moments, $d_{1}$ is the horizontal distance from the camera to A’ at the first moment, $d_{2}$ is the horizontal distance from the camera to B′ at the second moment, Δl is the distance between the road coordinate points corresponding to the camera imaging points at two moments.

The obstacle detection method based on VIDAR conforms to the working principle of camera ranging. This method analyzes the height change between the three-dimensional obstacle and the road plane when the monocular camera moves with the loaded vehicle and takes the target obstacle in the two adjacent frames obtained when the monocular camera moves as the detection object. It is assumed that the first imaging point of the obstacle on the two-dimensional imaging plane is A (see Figure 7). Because the camera moves with the vehicle, the axis in the two-dimensional imaging plane moves from $y_{1}$ to $y_{2}$. The imaging point corresponding to the same position of the second frame of the obstacle is B, and the imaging points A and B in the two frames correspond to points A′ and B′ on the road plane. The horizontal distance from the camera to A′ is $d_{1}$, and the horizontal distance from the camera to B′ is $d_{2}$. From Equation (7), the relation is $d_{1}=d_{2}+\Delta d+\Delta l$. If Δl=0, it is proved that the obstacle has a certain height, and the inertial measurement unit can be used to obtain the static obstacle.

The horizontal distance from the camera to the vertex of the obstacle at the previous moment is $S_{1}$. The distance that the camera moves at two moments is Δd, and the distance between the road coordinate points corresponding to the camera imaging points at two moments is Δl. Then,

(7) $$\left\{\begin{array}{c} d_{2}=d_{1}-\Delta l-\Delta d\\ S_{2}=S_{1}-\Delta d \end{array}\right.$$

According to the trigonometric function relation and Equation (7) of the right triangle, we can obtain

(8) $$\left\{\begin{array}{c} \frac{h_{v}}{h}=\frac{d_{1}-S_{1}}{d_{1}}\\ \frac{h_{v}}{h}=\frac{d_{2}-S_{2}}{d_{2}} \end{array}\right.$$

Substituting Equation (7) into Equation (8) yields

(9) $$S_{1}\Delta d+S_{1}\Delta l-d_{2}\Delta d-\Delta d^{2}-\Delta d\Delta l=0$$

Simplifying Equation (9), we obtain

(10) $$\frac{S_{1}}{d_{1}}=\frac{\Delta d}{\Delta d+\Delta l}$$

The height measurement equation for static obstacles can be obtained by bringing Equation (10) into Equation (8):

(11) $$h_{v}=\frac{h\times \Delta l}{\Delta l+\Delta d}$$

#### 3.2.3. Dynamic Obstacle Detection

**Figure 8:**
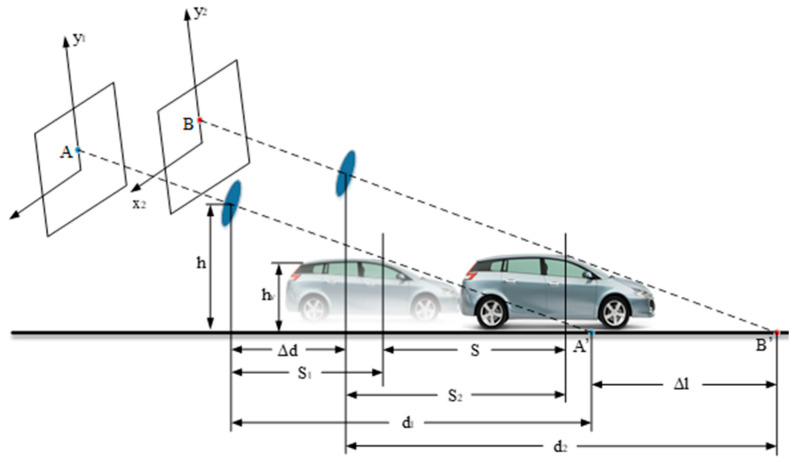
Schematic diagram of dynamic obstacle imaging, where $S_{1}$ is the horizontal distance from the camera to the vertex of the obstacle at the previous moment, $S_{2}$ is the horizontal distance from the camera to the same vertex of the obstacle in the following moment, h is the installation height of the camera, $h_{v}$ is the height of the obstacle, Δd is the distance that the camera moves at two moments, $d_{1}$ is the horizontal distance from the camera to A′ at the first moment, $d_{2}$ is the horizontal distance from the camera to B′ at the second moment, Δl is the distance between the road coordinate points corresponding to the camera imaging points at two moments, S is the moving distance of the target obstacle.

When the obstacle moves horizontally along the specified direction of the road (see Figure 8), the horizontal distance from the camera to the vertex of the obstacle at the previous moment is $S_{1}$, and the horizontal distance from the camera to the same vertex of the obstacle at the next moment is $d_{1}$,$d_{2}$. The relationship between $S_{1}$ and $S_{2}$ is

(12) $$\left\{\begin{array}{c} d_{2}=d_{1}+\Delta l-\Delta d\\ S_{2}=S_{1}+S-\Delta d \end{array}\right.$$

According to the trigonometric function relation and Equation (12) of the right triangle, we can obtain

(13) $$\left\{\begin{array}{c} \frac{h_{v}}{h}=\frac{S}{S+S_{1}}\\ \frac{h_{v}}{h}=\frac{d_{2}-S_{2}}{d_{2}} \end{array}\right.$$

Substituting Equation (12) into Equation (13) yields the following equation:

(14) $$S_{1}\times (\Delta l-\Delta d)=d_{1}\times (S-\Delta d)$$

The height measurement of dynamic obstacles can be obtained by simplifying Equation (14) as follows:

(15) $$h_{v}=\frac{(\Delta l-S)\times h}{\Delta l-\Delta d}$$

$\Delta l=\frac{h\times S-h_{v}\times \Delta d}{h-h_{v}}$ can be calculated by Equation (15). Since the position change of the obstacle in the two frames before and after the moving condition is not zero, that is, Δl≠0, the dynamic obstacle can be identified under any condition that does not conform to $h\times S=h_{v}\times \Delta d$. It is proved that monocular camera and inertial measurement unit can be used to detect obstacles. By tracking and calculating the position of feature points, the time consumption for detecting obstacles is reduced.

### 3.3. Data Interaction Based on UDP Protocol

In view of the fact that the roadside unit cannot identify the generalized obstacle of the approximate two-dimensional object, the fast road obstacle detection based on MSER can bypass the obstacle classification to detect the actual height of the generalized obstacle. This paper realizes the data sharing between the roadside unit and the vehicle unit through the User Datagram Protocol (UDP) and improves the uncertain obstacle information detected by the roadside unit. The roadside unit collects road environmental data through a camera connected to an RSU, which includes a roadside memory and communication module, a roadside controller, and a camera. The on-board camera is connected to an OBU and an on-board computing unit. The device on the on-board side includes an on-board communication module, an information acquisition device for obtaining vehicle operation information, and an on-board controller. The onboard controller is electrically connected to the onboard communication module and information acquisition device. The UDP protocol provides a connectionless and unreliable datagram service for terminal devices. UDP does not need to establish a connection before transmitting data. After the transport layer of the remote host receives the UDP message, there is no need to send a prompt statement that receives the data. Due to the need for real-time transmission of road information data between RSU and OBU, the volume of transmission data is small, such as the transmission of obstacle location information, images, etc. Therefore, this paper uses UDP to implement the data interaction between roadside units and vehicle units.

#### 3.3.1. Data Acquisition at RSU Side and OBU Side

**Figure 9:**
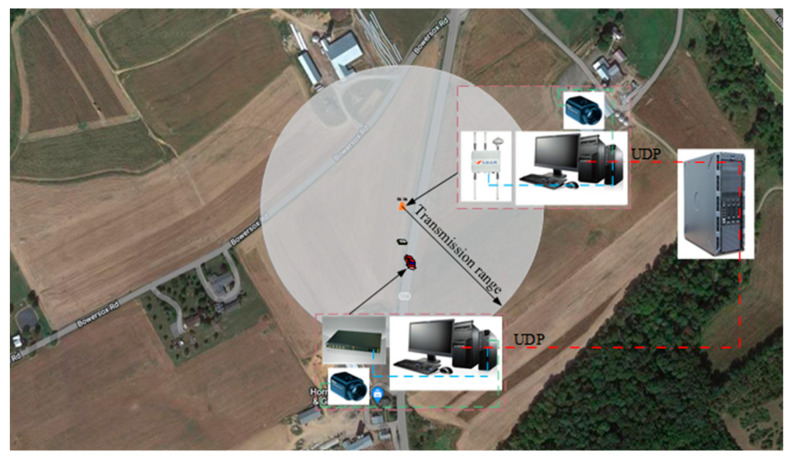
Data interaction structure diagram based on UDP, where the orange icon represents the placement position of roadside units.

The prerequisites for data interaction are accurate acquisition of target obstacle information and a reliable communication protocol. This subsection elaborates the process of environmental data acquisition at the roadside unit side and the vehicle unit side to provide data support for the process of data interaction. Figure 9 shows the schematic diagram of UDP-based data interaction.

#### 3.3.2. UDP Protocol Transmission UDP Adopts a Connectionless Mode

**Figure 10:**
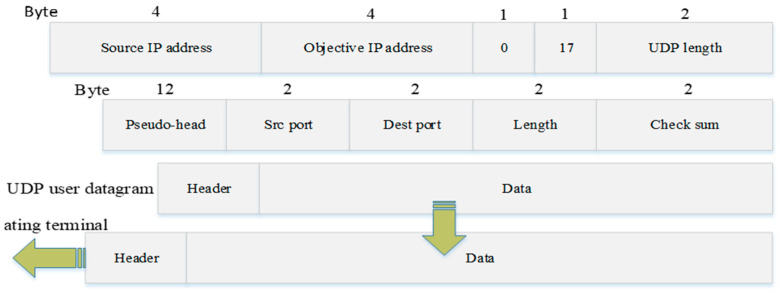
UDP message format.

UDP uses a connectionless mode. The messages sent by the sender do not have the function of controlling the data byte order, and there is no handshake mechanism between the receiver and the sender. In this case, the message order may be confused, and packet loss may occur. With the development of network technology, the packet loss rate of UDP transmission is very low, especially in the short distance transmission area. Compared with TCP, the transmission efficiency is high, and it is more suitable for data transmission in smaller areas. The UDP message format is shown in Figure 10. The UDP message has no guaranteed fields for reliability, flow, and order control, and the reliability issue of communication will be guaranteed by the application layer protocol. UDP has no flow control and error control mechanism, so there is no window check link. In the UDP processing queue, the queue opened by the client is identified by the ephemeral port number, and the queue works as long as the process is running and is revoked when the process terminates. The client specifies the data source port number in the request use process. Messages are sent to the outgoing queue, and UDP takes them out one by one and delivers them to the IP layer. When the message arrives at the client, UDP checks whether the port number corresponding to the destination port number field in that user data message exists in the queue. If that style queue exists, UDP places the received user data message at the end of that queue. If there is no such queue, UDP discards the user data message and requests the ICMP protocol to send an unreachable message to the server.

UDP is mainly used in networks that support the transmission of data between computers. The protocol enables multiple applications to send and receive data simultaneously at the same time by using port numbers to reserve their respective data transmission channels for different applications. The data sender sends UDP data messages out through the data source port, and the data receiver receives data through the destination port. Since UDP excludes the mechanism of reliable message delivery, the sorting process is handed over to the upper layer applications. Therefore, the execution time is reduced.

#### 3.3.3. UDP-Based Data Transfer

**Figure 11:**
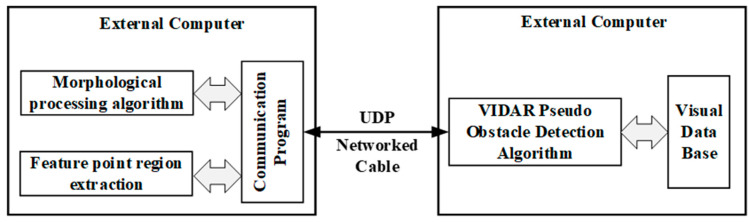
Communication port architecture diagram.

Since there is no ready-made program for UDP communication in the software, the communication client (RSU) program is written to receive and transmit data. The communication port architecture program is shown in Figure 11. A software program is run on an external computer at the RSU end, which provides morphological processing algorithms and feature area extraction algorithms. The obstacle location information generated by the feature area extraction algorithm is transmitted to the server (OBU) using the UDP protocol. A generalized obstacle detection algorithm based on VIDAR is provided on the external computer of the server. The communication port constructed using the UDP protocol transmits obstacle height information to the communication client.

## 4. Experiment and Result Analysis

For obstacle detection, a simulated real vehicle experiment is conducted in the laboratory. By setting some camera parameters, the complexity of the experiment can be reduced. Some camera internal parameters can be obtained according to the parameter descriptions in the product manual and after calibration. Table 1 shows some camera parameter settings for simulated real vehicle experiments.

### 4.1. RSU-End Obstacle Detection Test

**Figure 12:**
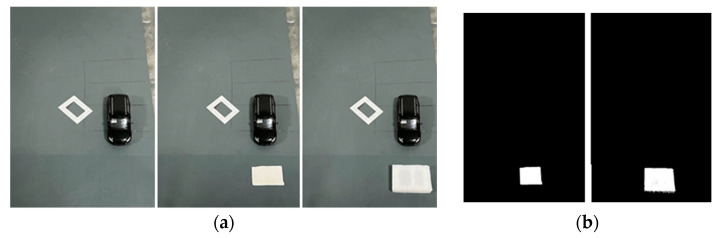

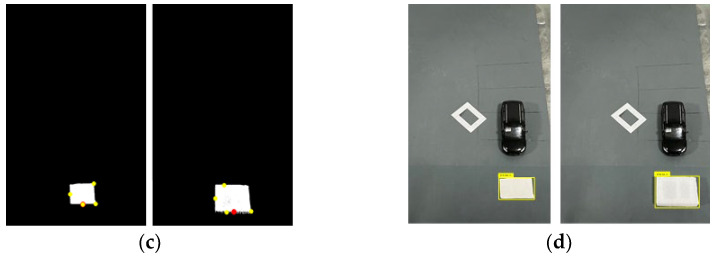
Obstacle extraction results based on the background difference method. (**a**) shows the before and after comparison of the initial background with the presence of obstacles; the first image is the experimentally selected background model $C_{n}$, and the second and third images are the current image frames with no height pseudo-obstacle and with height generalized obstacle selected, respectively. (**b**) is the obstacle region ${S_{P}}_{n}$ obtained by the two different height obstacles through the comparison image, using the background difference method processing. (**c**) is the coordinate position of the obstacle and the coordinate positioning map of the obstacle position region. (**d**) shows the obstacle detection result map.

After calibrating the roadside unit end camera, the video captured by the camera is divided into image sequences, and the background model and the current image frame are selected (see Figure 12a). It includes two generalized obstacles of different sizes and heights. The presence area of the obstacle can be obtained through the background difference method, and the position coordinates of the obstacle can be obtained through coordinate transformation. The specific experimental results are shown in Figure 12.

According to the coordinate conversion between the projection point of the road obstacle and the obstacle taken in the world coordinate system, it can be concluded that the coordinate of the pseudo-obstacle in the test is (40.25,60.34), and the coordinate of the generalized obstacle is (43.74,50.32). However, by comparing the background difference images of two obstacles, we cannot distinguish the authenticity of the obstacles. Therefore, the roadside unit using only the background difference method cannot provide completely true road information for the vehicle.

### 4.2. VIDAR-Based Vehicle-End Obstacle Detection Test

**Figure 13:**
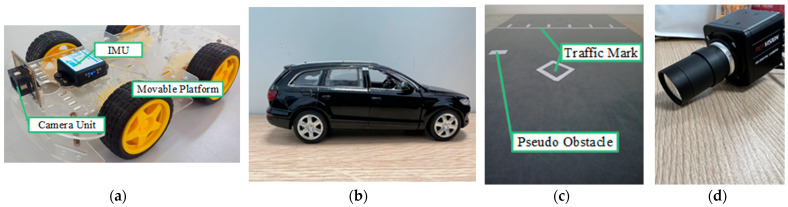
Indoor experimental equipment. (**a**) shows the mobile platform, camera unit, and IMU. (**b**) shows the vehicle scale model. (**c**) shows traffic signs and roadway wide obstacles. (**d**) shows the simulated roadside unit end camera.

In the experiment, the OV5640 image acquisition unit and JY61p IMU are installed on the vehicle-like mobile platform (see Figure 13a). Obstacles were simulated using a 1:70 scale model with a real vehicle (see Figure 13b). Traffic signs and road obstacles are replaced by paper attached to the experimental platform (see Figure 13c).

**Figure 14:**
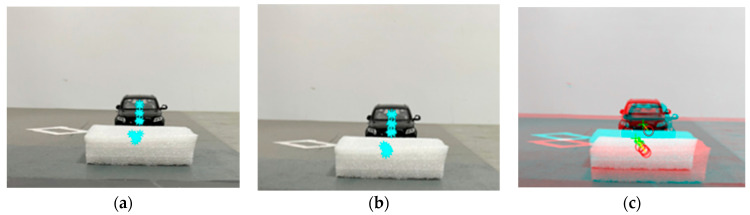
MSERs (Maximally stable extremal regions space) feature region extraction. (**a**) is the obstacle image at moment t=0, and (**b**) is the obstacle image at moment t=2. Additionally, (**c**) is the region matching image before and after the two moments, where the red region and o are the center of mass of MSERs and MSERs in the image at moment t=0, and the cyan region and + are the center of mass of MSERs and MSERs in the image at moment t=2.

In the vehicle-side obstacle detection, the camera pose is solved by P3P, and the horizontal distance Δd=2.00 cm of Δt=2 s in the obstacle period of the two images is calculated by acceleration data. The acceleration and angular acceleration data of the vehicle-like mobile platform can be obtained by the inertial measurement unit. In the experiment, the fast image region matching method based on mean square error is used to process the images at time t=0 and time t=2 (see Figure 14). The centroids of the extracted seven matching regions are taken as feature points (see Figure 14a,b).

**Figure 15:**
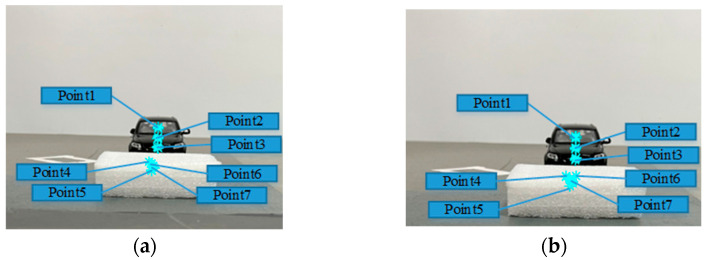
Obstacle feature point location detection result figure, where (**a**) is the location of the feature point located in the image at moment t=0, and (**b**) is the location of the feature point located in the image at moment t=2.

The feature points are extracted according to the two frames of images obtained by the vehicle camera (see Figure 15), and the horizontal distance between each feature point and the camera is detected by the monocular ranging algorithm. The results are shown in Table 2.

**Figure 16:**
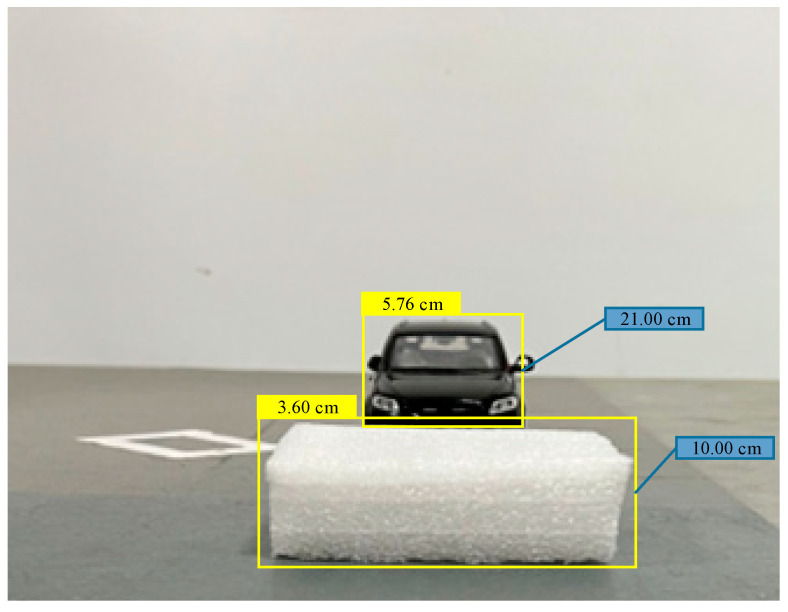
Obstacle area division result map (where the yellow box is the detected obstacle area and the height information of that obstacle is displayed on its box, and the data in the blue box are the distance from the obstacle to the camera).

**Figure 17:**
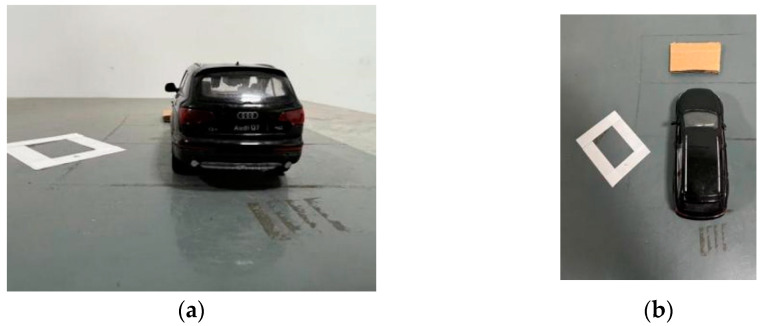
Example of indoor experiments in the presence of target occlusion, where (**a**) shows the road environment interface detected by the on-board camera, and (**b**) shows the road environment interface detected by RSU.

The calculation results show that the mean square error corresponding to the feature points is defined as the obstacle area, and the obstacle area is marked as the MSERs area. Feature points 1, 2, and 3 are feature points on the vehicle model, and the rest are feature points on the target generalized obstacle. The center point of the intersection line between the bottom boundary of the obstacle area and the road is taken as the ranging point of the obstacle, and the pinhole camera model is used to calculate the distance from the obstacle to the camera. According to Figure 16, the height of the target generalized obstacle detected in the experiment is 10.00 cm, the height of the vehicle model is 21.00 cm, the distance between the image acquisition unit and the target generalized obstacle is 3.60 cm, and the distance from the vehicle model is 5.76 cm. However, this method cannot solve the problem of the target obstacle detection out of view caused by object occlusion (see Figure 17).

### 4.3. Experiment of Obstacle Detection Based on RSU and Vehicle Camera

**Figure 18:**
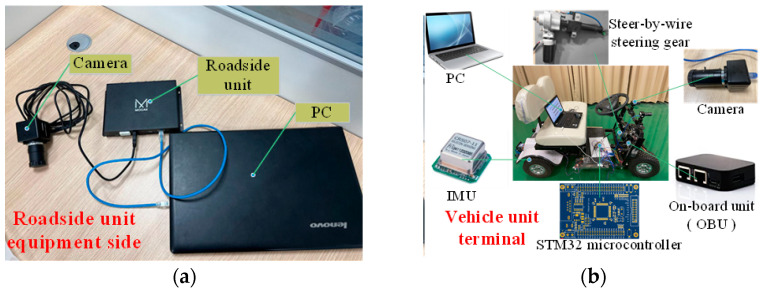
Overall equipment diagram for data interaction, where (**a**) shows the overall equipment at the equipment side of the roadside unit, and (**b**) shows the overall equipment at the equipment side of the vehicle.

**Figure 19:**
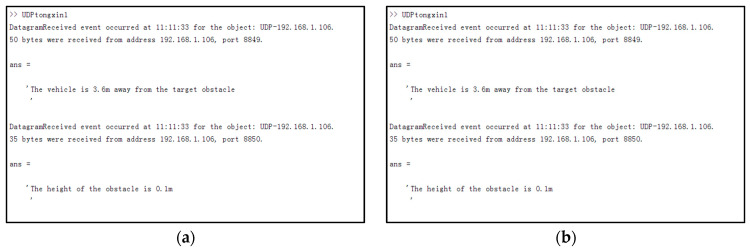
Diagram of information transmission results at both ends. (**a**) shows the data information received from MATLAB at the roadside unit device end to MATLAB at the vehicle device end for processing, which includes the obstacle height and the result data of the vehicle distance from the target obstacle, and (**b**) shows the data information received from MATLAB at the vehicle device end to MATLAB at the roadside unit device end, which includes the obstacle coordinate position and the obstacle discrimination information.

This experiment realizes obstacle detection based on the RSU and vehicle camera through data interaction. Figure 18 describes the entire device structure. Figure 19 shows the communication results between the two ends, showing the data content of the data interaction between the two ends. The experiment consisted of four elements: an obstacle, a pure electric vehicle equipped with a vehicle unit and a vision sensor, an RSU device, and two laptop computers serving as RSU and vehicle unit servers. The RSU-end MATLAB transmits the detected target obstacle position coordinate $\left(43.74,50.32\right)$ to the vehicle-unit-end MATLAB through the UDP protocol (see Figure 19b). The equipment model and whether the experimental environment meets the experimental requirements are shown in Table 3. OBU has a USB interface, and the RSU supports DSRC (Dedicated Short Range Communications) because of a USB interface. Since OBU and RSU do not share a common wireless technology, they cannot communicate wirelessly. The OBU installed on a pure electric vehicle has both of these technologies and runs WiFi access point (AP) services. The RSU and laptop are connected by a standard ethernet cable.

The operation of the experiment is as follows. Firstly, the road information is detected in real time by the RSU camera, and the obstacle position information in the road is marked. The obstacle type includes the identifiable obstacle type (vehicle, pedestrian, etc.) and the generalized obstacle type (plank, foam box, etc.). The three-dimensional data and position of the identifiable obstacle are 3D framed in the graph, and the specific-type label is marked. For the generalized obstacle type, only the obstacle position coordinates are obtained, and the large-type label is marked as an obstacle. When the vehicle is close to the generalized obstacle, the on-board camera opening request is sent, and the on-board sensor obtains the road information in real time. The OBU collects the environmental data (obstacle position/distance between the car and the obstacle, etc.) in the sensor hardware and continuously scans the wireless media to search the WiFi AP client. Meanwhile, when the vehicle follows the designated route to the obstacle detection range, and the OBU and RSU enter the communication range, the OBU transmits the collected data to the RSU. A UDP protocol is established between the RSU and OBU to transmit sensor data and obstacle information. After that, the obstacle detection method based on VIDAR is used to quickly and accurately detect the height of the generalized obstacle. The obstacle type is judged by judging the height difference $d_{z}=h_{v}-h_{c}$ between the height of the generalized obstacle and the maximum passing height of the vehicle chassis. When $d_{z}>0$, the generalized obstacle is a real obstacle, the tag and the block diagram are unchanged, and the 3D block diagram is made according to its height and the length and width data of the obstacle detected by the RSU camera. When $d_{z}<0$, the generalized obstacle is defined as an uninfluenced obstacle, and the block diagram of the obstacle in the RSU camera detection interface can be removed. Finally, all the received and processed data are stored in the database for integration to obtain complete road information. UDP packets are implemented by setting the number of clients, IP address, and the longest time interval to send in the MATLAB data interface port station. After setting the data port, the client sends the measurement point information to the IP address specified in the UDP packet. A data-receiving program based on the UDP object creation function was written in MATLAB. It can read formatted data from the server MATLAB. Then, the data is saved to the MATLAB workspace to call other programs. The command to create a UDP object is ‘udpA=udp(IPB,portB,‘LocalPort’,portA)’. ‘IPB’ and ‘portB’ are the IP address and port number of the client data port, while ‘portA’ is the local port number. By connecting the same WiFi between the client and the server, the data transmission environment under the same network at both ends is realized. At the same time, the callback function display when the udpA receives the data packet is set, and the data type to be sent and the location of the data sending and receiving ports are determined. The simple transmission of experimental data in the MATLAB environment can be realized.

**Figure 20:**
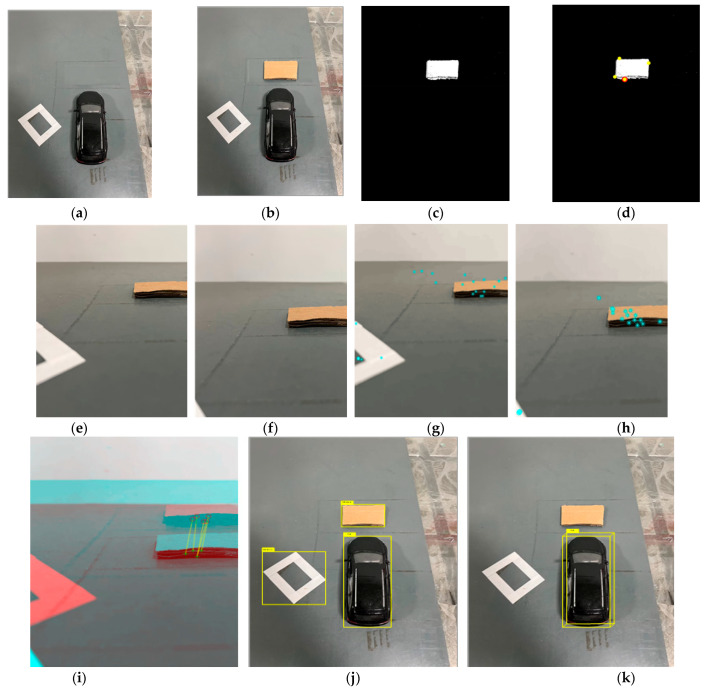
Test process of obstacle detection method based on roadside unit and vehicle mounted camera. (**a**) is the initial background image, (**b**) is the current frame image, (**c**) is the obstacle area SPn obtained by the background difference method processing, (**d**) is the coordinate position of this obstacle obtained, (**e**) is the obstacle image at the moment t=0, (**f**) is the obstacle image at the moment t=2, (**g**) is the feature point extraction of the obstacle image at the moment t=0, (**h**) is the feature point extraction of the obstacle image at the moment t=2, and (**i**) is the global environment information at the camera end of the roadside unit and each marker label. (**j**) is the corrected global environment information after the obstacle detection method based on the roadside unit and the vehicle camera. (**k**) shows the area matching images before and after the two moments.

The scene shown in Figure 20 is taken as the test scene. Firstly, the initial background image and the current frame image are obtained by the RSU camera (see Figure 20a,b). The background difference image $E_{n}$ is obtained by the background subtraction method (see Figure 20c). According to the generalized obstacle coordinate position positioning method, the target generalized obstacle coordinate is (43.74,50.32)(see Figure 20d). Due to the occlusion of the front vehicle, the vehicle-mounted unit camera cannot detect the target generalized obstacle (the diamond-shaped landmark is a pseudo-obstacle, and the obstacle of the occluded vehicle model can be deduced by the vehicle-side camera using the obstacle detection method based on VIDAR; the obstacle height is 1.69 m). The target generalized obstacle information can be detected by moving the vehicle (see Figure 20e,f). The obstacle detection method based on VIDAR is used to detect the target generalized obstacle, and the distance from the vehicle to the obstacle can be determined to be 3.6 m. According to Table 4 and Equation (10), the height of the obstacle can be deduced, and the height of the obstacle obtained can be determined by arithmetic average to be 0.1 m. According to the minimum height threshold of the vehicle chassis set by the RSU, the target obstacle is judged to be an unaffected obstacle, the information is transmitted to the vehicle equipment end (see Figure 19a), and the obstacle block diagram is eliminated (see Figure 19b).

## 5. Analysis of the Effect of Obstacle Detection Method Based on the Fusion of RSU and Vehicle Camera

**Figure 21:**
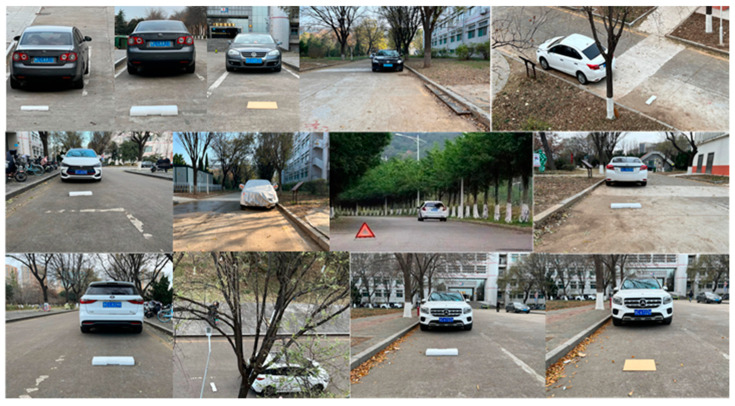
Partial dataset, these images are partially in line with the scenario set in this article.

The campus road environment was recorded by the SONY IMX179 camera as well as the roadside unit camera, and the camera pose data were recorded by the HEC295 IMU. The pose of the camera at the roadside unit end was fixed, and the image data and camera pose data were processed by YOLOv5, the roadside unit-based obstacle detection method, the VIDAR-based vehicle-end obstacle detection method, and the obstacle detection method based on the fusion of roadside unit and vehicle cameras, respectively. The number of datapoints in the dataset is 7356. Figure 21 shows some of the data in the dataset. The results compare and analyze the accuracy and detection speed of the four methods.

### 5.1. Detection Accuracy Analysis

In the accuracy analysis of remote sensing and pattern recognition, detection accuracy (DT), true accuracy (RT), and overall accuracy (OT) are usually used to judge or compare the accuracy of methods [32,33,34]. In this paper, the number of obstacles detected as obstacles but not actually obstacles or generalized obstacles is denoted as $a_{i}$. The number of obstacles detected as obstacles and actually obstacles is denoted as $b_{i}$. The number of obstacles that are not detected by the detection method but are actually obstacles is recorded as $c_{i}$. The number of obstacles that are not detected as obstacles by the detection method and are not actually obstacles or generalized obstacles is denoted as $d_{i}$. The test result confusion matrix is shown in Table 5. By calculating the number of TC, FC, TN, and FN obstacles in two adjacent frames of images, the accuracy of the obstacle detection method based on RSU, the vehicle-end obstacle detection method based on VIDAR, and the obstacle detection method based on RSU and vehicle camera fusion are compared: $TC=\sum_{i=1}^{n}a_{i}$, $FC=\sum_{i=1}^{n}b_{i}$, $TN=\sum_{i=1}^{n}c_{i}$, $FN=\sum_{i=1}^{n}d_{i}$.

In the result analysis, detection accuracy (DT), true accuracy (RT), and overall accuracy (OT) are used as the evaluation indexes of three generalized obstacle detection methods.

(16) $$OT=\frac{FN+FC}{TC+FC+TN+FN}$$

(17) $$DT=\frac{FC}{TC+FC}$$

(18) $$RT=\frac{FC}{FC+TN}$$

The obstacle detection method based on RSU, the vehicle-side obstacle detection method based on VIDAR, and the obstacle detection method based on the fusion of RSU and vehicle camera are shown. The confusion matrix of obstacle detection results is shown in Table 6.

As can be seen from Table 6, the RT and DT of the generalized obstacle detection method based on the fusion of the RSU and vehicle camera are higher than the other three methods. This is because in a complex traffic environment, some obstacles can move away from the camera or move quickly. The obstacle detection method based on the fusion of the RSU and vehicle camera can detect and judge the type of obstacle in advance by RSU. When the vehicle moves to the detection range of machine vision, the vehicle can obtain the target obstacle information through the vehicle camera and then transmit it to the RSU for road information storage through the UDP protocol to provide road environment information for subsequent vehicles.

**Figure 22:**
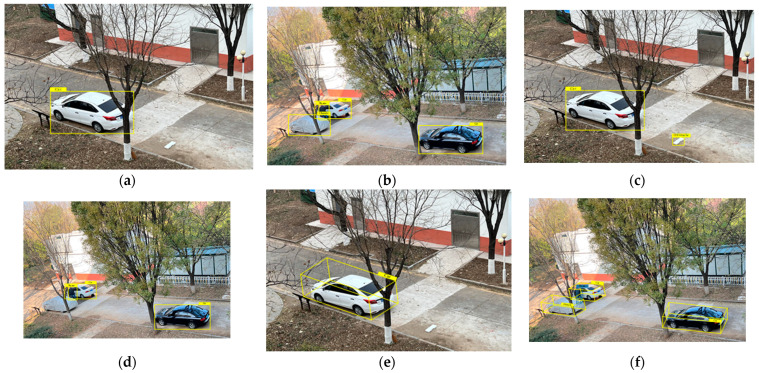
Comparison graph of obstacle detection results. (**a**,**b**) show the results of the VIDAR-based obstacle detection method. (**c**,**d**) show the results of the roadside-unit-based obstacle detection method. (**e**,**f**) show the result of obstacle recognition after processing the obstacle detection method based on the roadside unit and the vehicle-mounted camera.

The results show that the VIDAR-based obstacle detection method is more accurate than the YOLOv5-based generalized obstacle detection method. This is because the VIDAR-based obstacle detection method can define the generalized obstacle by deriving the obstacle height. Both the roadside unit and vehicle camera fusion-based obstacle detection method and the VIDAR-based obstacle detection method have a higher accuracy index than the roadside-unit-based obstacle detection method in vehicle obstacle detection. The roadside-unit-based obstacle detection method has a greater impact on the detection of the roadside unit due to its detection viewpoint and the location of the obstacle. For example, the obstacle is in the area directly below the camera of the roadside unit, or the broad obstacle exists within its field of view. These situations can reduce the accuracy of the roadside-unit-based obstacle detection method. In addition, the roadside-unit-based obstacle detection method can only detect trained targets. If the obstacle in the test is a generalized obstacle, the roadside-unit-based obstacle detection method will not be able to confirm the type of obstacle detected. The method can only define such obstacles as “obstacle class”. The further detection of such obstacles is required to determine the authenticity of the obstacles. The VIDAR-based obstacle detection method is capable of detecting undefined broad obstacles. However, the method has a bias in detecting the target obstacle when the vehicle speed is fast. Therefore, the use of roadside-unit-based and vehicle camera fusion obstacle detection not only can improve the speed of machine learning but also can improve the accuracy of obstacle recognition. Figure 22 shows the detection comparison of the three methods. The experimental results show that the method is able to achieve the detection and classification function of broad obstacles in the road.

### 5.2. Detection Speed Analysis

The VIDAR-based obstacle detection method and the roadside unit and vehicle-mounted camera fusion-based obstacle detection method are used to pre-process the images and camera pose data acquired before and after the vehicle movement, respectively. The detection speed and running time of the four methods are compared based on the calculated total time required to detect all obstacles in the images. The average detection time and running time of the four detection methods are shown in Table 7.

As can be seen from Table 7. The detection speed of the VIDAR-based obstacle detection method and the roadside unit and vehicle-mounted camera-based obstacle detection method is faster than that of the roadside-unit-based obstacle detection method. This is because the method eliminates the process of elliptical region fitting and feature point detection with SIFT or ASIFT. The operation of the obstacle detection method based on the fusion of roadside units and on-board cameras is slower than that of the roadside-unit-based obstacle detection method. This is because the joint method has to interact with data through communication between the two ends, during which some transmission time is lost. However, because of the poor detection effect of the roadside unit, the training dataset for this type of obstacle needs to be added later for training. Although data interaction is required between the roadside device side and the vehicle device side, the overall time required is less compared to the roadside-unit-based obstacle detection method. The same is true for the YOLOv5-based obstacle detection method, because a large number of samples are trained in advance, making it faster in detection time than the method proposed in this paper, and the system built is more mature. The method proposed in this paper only uses real-time MATLAB software code to implement the obstacle detection, and the subsequent system can be built to further improve the running time and its detection speed. Therefore, the method proposed in this paper can provide the over-the-horizon distance through the vehicle. Although it is slower than the machine learning method in terms of obstacle recognition speed, it omits the sample training process, and the overall decision time is faster than the rest of the methods.

## 6. Conclusions

In this paper, an obstacle detection method based on the fusion of the roadside unit and vehicle-mounted camera is proposed. In the fast image region-matching method for vehicle-side obstacle detection, the process of ellipse region fitting and the SIFT or ASIFT extraction of feature points are omitted. The MSER-based fast image area-matching method is combined with the vision IMU-based obstacle detection method to bypass obstacle classification. The method reduces the spatio-temporal complexity of road environment perception and improves the speed and accuracy of image matching. The background difference method is applied at the roadside unit end to extract the generalized obstacles, and special points are used to localize the target obstacles. The horizontal distance from the camera to the target obstacle and the height of the target obstacle are derived by converting the position coordinates of the obstacle in the world coordinate system and the imaging coordinate system. The theoretical feasibility of the combined detection method of the monocular camera and inertial measurement unit with the roadside unit is clearly illustrated. In order to realize the data interaction between the equipment side of the roadside unit and the vehicle equipment side, this paper transmits the road environment data obtained from both ends through the UDP protocol.

The detection process of the pseudo-obstacle detection method based on the fusion of the roadside unit and vehicle-mounted camera is illustrated through indoor generalized obstacle detection experiments and two-end data interaction experiments. The VIDAR-based obstacle detection method, the roadside unit-based obstacle detection method, the YOLOv5 obstacle detection method, and the method proposed in this paper are compared in the outdoor test, and the test results are compared with DT, RT, and OT as evaluation indexes. The results show that the method has high accuracy, and the reasons for the high accuracy are analyzed. The obstacle detection speed of the four methods is compared, and the results show that the method has a faster processing speed, and the reasons for the faster processing speed are analyzed.

In this paper, a methodological innovation is made in the detection of generalized obstacles, and a generalized obstacle detection method based on the fusion of roadside units and vehicle-mounted cameras is used to achieve the accurate detection of generalized obstacles. However, there are shortcomings in data interaction, especially in data loss and data security protection. Subsequent research directions will be devoted to the design of road environment data interaction methods that can guarantee low data loss rate and high data security conditions.

## Figures and Tables

**Table 1:** Visibility prediction test data table.

Camera Parameter Name	*Parameter Symbol*	*Numerical Value*	*Company*
Pixel size of the photosensitive chip	p	1.4	μm
Camera mounting height	h	6.572	cm
Camera pitch angle	γ	0.132	rad
Effective focal length of camera	f	6.779	mm
Minimum height of vehicle chassis	h_c_	14	cm

**Table 2:** Calculation results of *d*_1_, *d*_2_, ∆*l*, and *h_v_*.

Feature Point	*d*_1_/cm	*d*_2_/cm	∆*d*/cm	∆*l*/cm	*h_v_*/cm
1	20.05	19.21	2.00	1.16	22.21
2	19.99	19.07	2.00	1.08	20.89
3	19.97	18.98	2.00	1.01	19.90
4	15.77	15.55	2.00	1.78	10.05
5	15.75	15.59	2.00	1.84	10.02
6	15.74	15.59	2.00	1.85	9.98
7	15.73	15.62	2.00	1.89	9.95

**Table 3:** Test conditions and equipment model.

Name	Specific Description
RSU-end laptop	Intel Core i5-6200U (Lenovo Co., Ltd., Beijing, China)
On-board unit-end laptop	Intel Core i7-6500U (Lenovo Co., Ltd., Beijing, China)
Camera	SONY IMX179 (Kexun Limited, Hong Kong, China)
RSU	MOKAR I-Classic (Huali Zhixing, Wuhan, China)
IMU	HEC295 (Weite Intelligent Technology Co., Ltd., Shenzhen, China)
STM32 single chip microcomputer	STM32F103VET6 CAN RS485 (Jiaqin Electronics, Shenzhen, China)
OBU	VSC-305-00D (Huali Zhixing, Wuhan, China)
System support	Windows7/10
Applicable operating temperature	24 °C
IPv4 address of RSU side	192.168.1.103
IPv4 address of on-board equipment	192.168.1.106

**Table 4:** Calculation results of *d*_1_, *d*_2_, ∆*l*, and *h_v_*.

Feature Point	*d*_1_/cm	*d*_2_/cm	∆*d*/cm	∆*l*/cm	*h_v_*/cm
1	10.93	10.80	2.00	1.87	9.97
2	11.02	10.87	2.00	1.85	9.99
3	11.02	10.83	2.00	1.81	10.02
4	11.01	10.82	2.00	1.81	10.02

**Table 5:** Confusion matrix of detection result.

		Actual Obstacle Category	
		True	None or False
**Detection result**	**True**	$b_{i}$	$a_{i}$
	**None or false**	$c_{i}$	$d_{i}$

**Table 6:** Obstacle detection result confusion matrix based on RSU.

Detection Method			Actual Obstacle Category		Identification Accuracy		
			True	None or False	DT	RT	OT
Obstacle detection method based on VIDAR	Detection result	True	6828	106	0.984	0.980	0.968
		None or false	140	282			
RSU-based obstacle detection method		True	5976	89	0.985	0.826	0.817
		None or false	1258	33			
Obstacle detection method based on RSU and vehicle camera		True	6934	13	0.998	0.976	0.991
		None or false	164	245			
YOLOv5		True	6798	104	0.985	0.974	0.961
		None or false	180	274			

**Table 7:** Average detection time of three methods.

Method	Obstacle Detection Method Based on VIDAR	Obstacle Detection Method Based on RSU	RSU and Vehicle Camera Integration	YOLOv5
Detection Time/s	0.364	0.371	0.367	0.359

## Data Availability

Not applicable.
